# Structural features of free *N*-glycans occurring in plants and functional features of de-*N*-glycosylation enzymes, ENGase, and PNGase: the presence of unusual plant complex type *N*-glycans

**DOI:** 10.3389/fpls.2014.00429

**Published:** 2014-09-04

**Authors:** Megumi Maeda, Yoshinobu Kimura

**Affiliations:** Functional Glycobiochemistry, Department of Biofunctional Chemistry, Graduate School of Environmental and Life Science, Okayama UniversityOkayama, Japan

**Keywords:** free *N*-glycans, PNGase, ENGase, knockout plant, glycochaperone

## Abstract

Free *N-*glycans (FNGs) are present at micromolar concentrations in plant cells during their differentiation, growth, and maturation stages. It has been postulated that these FNGs are signaling molecules involved in plant development or fruit ripening. However, the hypothetical biochemical and molecular function of FNGs has not been yet established. The structure of FNGs found ubiquitously in plant tissues such as hypocotyls, leaves, roots, developing seeds, or fruits can be classified into two types: high-mannose type and plant complex type; the former, in most cases, has only one GlcNAc residue at the reducing end (GN1 type), while the latter has the chitobiosyl unit at the reducing end (GN2 type). These findings suggest that endo-β-*N-*acetylglucosaminidase (ENGase) must be involved in the production of GN1 type FNGs, whereas only peptide:*N-*glycanase (PNGase) is involved in the production of GN2 type FNGs. It has been hypothesized that cytosolic PNGase (cPNGase) and ENGase in animal cells are involved in the production of high-mannose type FNGs in order to release *N*-glycans from the misfolded glycoproteins in the protein quality control systems. In the case of plants, it is well known that another type of PNGase, the acidic PNGase (aPNGase) is involved in the production of plant complex type FNGs in an acidic organelle, suggesting the de-*N-*glycosylation mechanism in plants is different from that in animal cells. To better understand the role of these FNGs in plants, the genes encoding these *N*-glycan releasing enzymes (ENGase and PNGase) were first identified, and then structure of FNGs in ENGase knocked-out plants were analyzed. These transgenic plants provide new insight into the plant-specific de-*N-*glycosylation mechanism and putative physiological functions of FNGs. In this review, we focus on the structural features of plant FNGs, as well as functional features of cPNGase/ENGase and plant specific PNGase, and putative functions of FNGs are also discussed.

## STRUCTURAL FEATURES OF *N-*GLYCANS LINKED TO THE PLANT *N-*GLYCOPROTEINS

**FIGURE 1:**
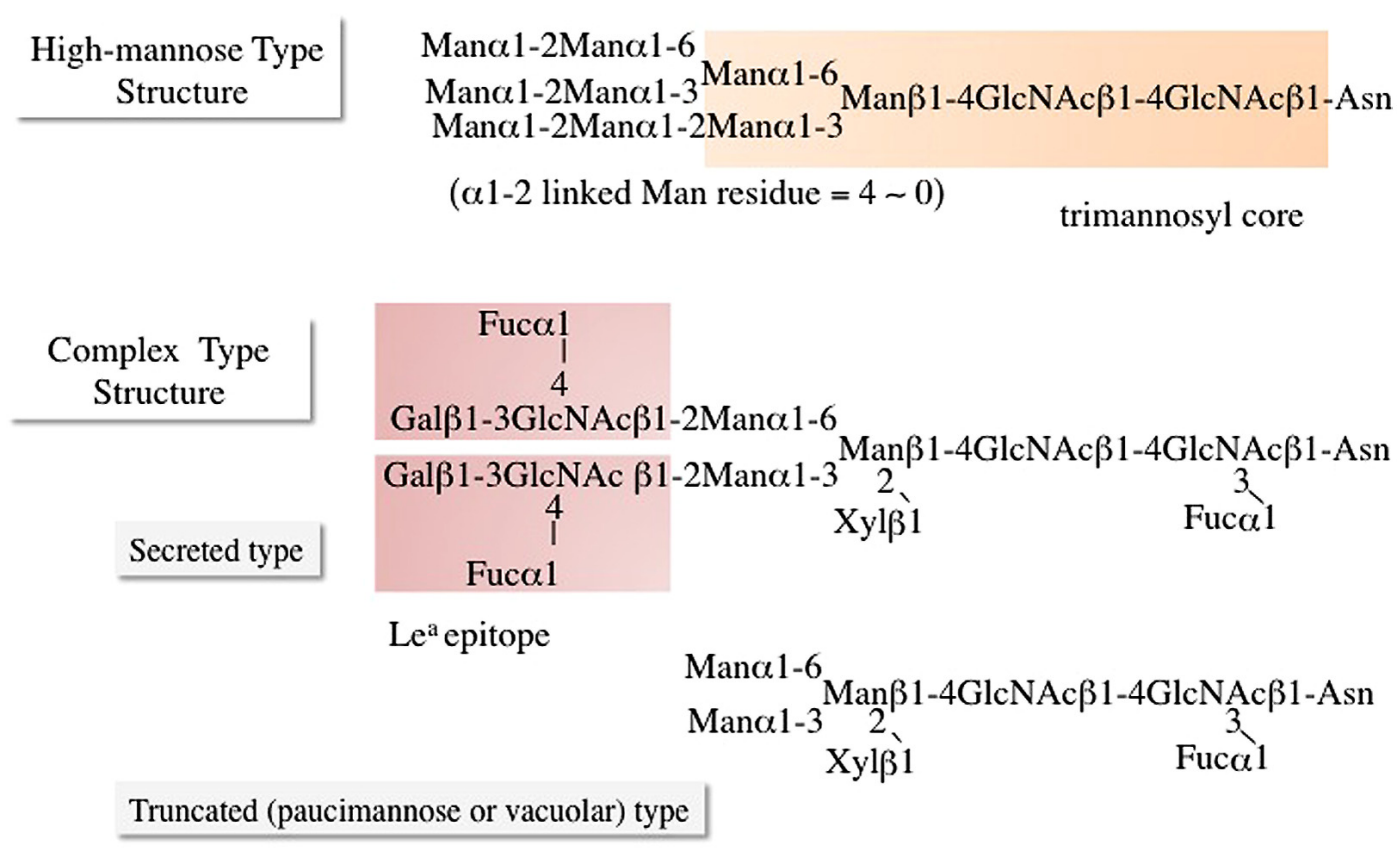
**Typical structures of plant *N*-glycans: high-mannose type structure and two kinds of complex type *N*-glycans**.

**FIGURE 2:**
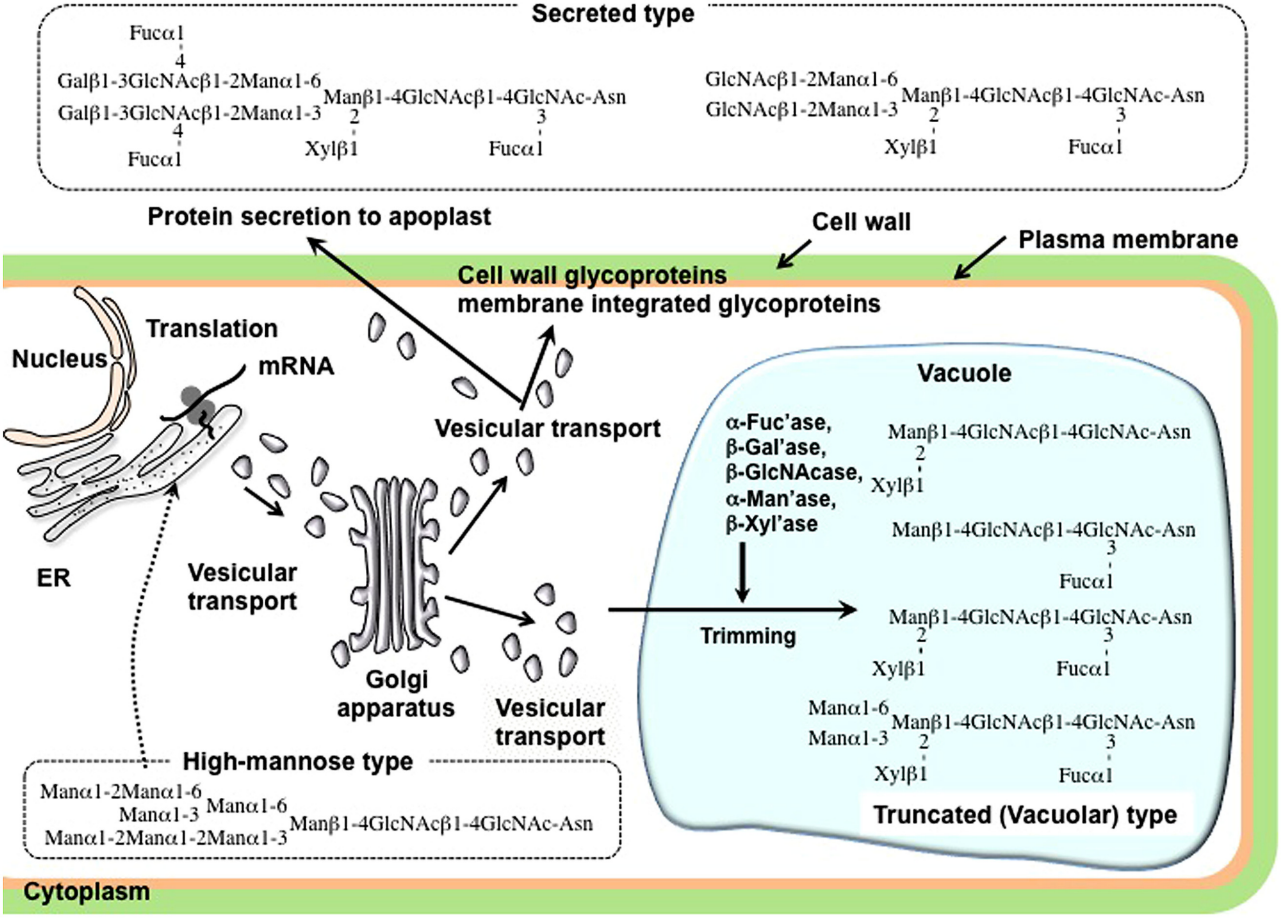
**Schematic representation of the possible processing pathway of plant *N*-glycans**.

It is well known that almost all the secreted-type proteins produced in eukaryotic cells are *N-*glycosylated, suggesting that the *N-*glycosylation of proteins and the subsequent modification of the glycan moiety are ubiquitous and pivotal biological process in eukaryotes, especially the multicellular organisms (Lerouge et al., 1998; Kobata, 2007). In the protein quality-control mechanism working in the endoplasmic reticulum (ER), it is believed that these *N-*glycans are linked to the nascent glycoproteins in the ER and play a critical role as ligands for calnexin or calreticulin, molecular chaperons that assist the protein-folding system in the ER (Suzuki et al., 2007). In plant cells, for example, deletion of ER α-glucosidase I, an enzyme that hydrolyzes α-1,3-glucosidic linkages in the nascent and premature *N-*glycans and controls the interaction of the unfolded nascent glycoproteins and the molecular chaperons, results in death (Boisson et al., 2001; Gillmor et al., 2002). *N-*Glycans that are biosynthesized in eukaryotic cells (regardless of animal, plant, insect, fungi, and yeast) share a common trimannosyl core structure [Manα1-6(Manα1-3)Manβ1-4GlcNAcβ1-4GlcNAc], but the final structure of these *N-*glycans varies by species. In the case of animals, plants, and insects, the *N-*glycan structures are classified into three subgroups: high-mannose type, hybrid type, and complex type depending on the location of additional sugar residues transferred to the trimannosyl core. The high-mannose type *N-*glycans are common in all eukaryotic cells, but the structural features of complex-type *N-*glycans vary widely depending on the species. The structural features of plant *N-*glycans are as follows: (1) the occurrences of β1,2-xylosyl (Xyl) residue linked to β1,4-mannosyl (Man) residue and α1,3-fucosyl (Fuc) residue linked to the reducing end *N-*acetylglucosaminyl (GlcNAc) residue, (2) the occurrence of Lewis a (Le^a^) epitope [Galβ1-3(Fucα1-4)GlcNAc] at the non-reducing end, and (3) the lack of *N*-acetylneuraminic acid residue and β1,4-linkage galactosyl (Gal) residue. The combination of α1,3-Fuc and β1,2-Xyl linked to the trimannosyl core is an outstanding characteristic of the plant complex type *N*-glycans. The most abundant complex type *N*-glycan linked to storage glycoproteins in seeds or vacuole-accumulated glycoproteins is Manα1-6(Manα1-3)(Xylβ1-2)Manβ1-4GlcNAcβ1-4(Fucα1-3)GlcNAc, which is sometimes described as a truncated-type, pauci-mannose-type, or vacuolar-type structure. Furthermore, the secreted plant glycoproteins sometimes carry large complex type *N*-glycans with Le^a^ epitope. The plant *N*-glycans harboring the Le^a^ epitope, which are sometimes referred to as secreted type structures, have been found in many foodstuffs and pollen allergens (Fichette-Lainè et al., 1997; Alisi et al., 2001; Wilson et al., 2001; Kimura et al., 2005; Maeda et al., 2005), although their physiological function in plants remains to be elucidated. The conversion of *N-*glycan structures from high-mannose type to complex type seems to play an important role in adaptation to environmental changes. It has been reported that the maturation of *N-*glycans to the complex type *N-*glycans in the Golgi apparatus is a prerequisite for the sufficient cell-wall formation under salt stress (Kang et al., 2008). Typical structures of *N-*glycans linked to plant glycoproteins are shown in **Figure 1**, and the possible processing pathway of plant *N-*glycans is outlined in **Figure 2**.

## STRUCTURAL FEATURES OF FREE *N-*GLYCANS IN PLANTS

**FIGURE 3:**
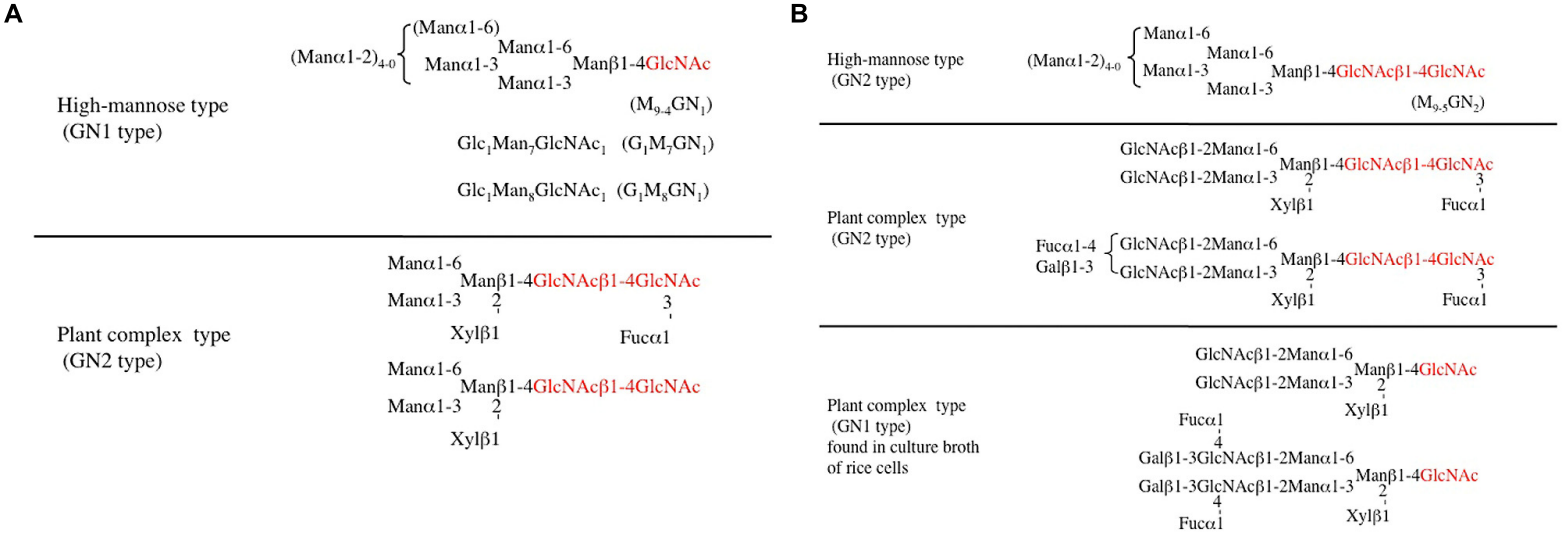
**Typical structures of free *N*-glycans (FNGs) found in plants**. **(A)** Free *N*-glycans found in the soluble fraction of rice cells (cytosol and vacuole). **(B)** Free *N*-glycans found in culture broth (extracellular space).

As the name implies, *N-*glycans are linked to a specific asparagine (Asn) residue in eukaryotic proteins, but free forms of these glycans, namely free *N-*glycans (FNGs), have been found in seedlings, stems, developing fruits, and culture cells (Priem et al., 1990b, 1993; Faugeron et al., 1997a,b; Kimura et al., 1997; Kimura and Matsuo, 2000; Kimura and Kitahara, 2000). Since similar FNGs have been found in animal cells, the occurrence of these FNGs is not just limited to plants. FNGs found in both plants and animals are classified into two types, GN1 type and GN2 type, based on the reducing terminal structure; GN1 type FNGs with one GlcNAc residue and GN2 type FNGs with GlcNAcβ1-4GlcNAc (*N-*acetylchitobiosyl unit). The structures of typical FNGs found in plants are summarized in **Figure 3**.

It is generally believed that FNGs are produced from the misfolded glycoproteins by cytosolic peptide:*N-*glycanase (cPNGase), which hydrolyzes the amide linkage in the glycosylated Asn residue, prior to degradation of the misfolded proteins by proteasomes in the cytosol. The disposal of misfolded/miss-associated proteins is known as ER-associated degradation (ERAD), and the resulting FNGs produced by cPNGase always belong to high-mannose type structure with the *N-*acetylchitobiosyl unit at their reducing ends (GN2 type FNGs). As for plant cPNGase, it was confirmed that the product of putative *Arabidopsis* PNGase gene when transformed in budding yeast facilitates the glycoprotein ERAD activity, suggesting that the gene product probably possesses PNGase activity and is likely involved in deglycosylation of misfolded glycoproteins (Diepold et al., 2007; Masahara-Negishi et al., 2012). In the cytosolic metabolism of FNGs released from misfolded proteins by cPNGase, GN2 type FNGs are rapidly modified by cytosolic endo-β-*N-*acetylglucosaminidase (cENGase), which hydrolyzes the glycosidic linkage in the *N-*acetylchitobiosyl unit to form the GN1 type FNGs (Suzuki and Funakoshi, 2006). Since almost all high-mannose type FNGs belong to the GN1 type, it is clear that ENGase is involved in the production of the high-mannose type FNGs found in plants, but there is no experimental evidence suggesting that such high-mannose type GN1-FNGs are exclusively generated from GN2 FNGs (cPNGase products) and not from the misfolded glycoproteins by the action of ENGase.

On the other hand, almost all plant complex type and truncated type FNGs belong to GN2 type, suggesting that only PNGase, but not ENGase, must be involved in the production. As we discuss later, the plant ENGase shows strong activity against high-mannose type *N-*glycans having Manα1–2Manα1–3Manβ1-unit, but no activity against plant complex and truncated structures having GlcNAcβ1-4(Fucα1-3)GlcNAc-unit at their reducing ends. It is, therefore, reasonable to conclude that almost all plant complex type FNGs have the GN2 type structure. Based on the biosynthesis or processing mechanism of *N-*glycans linked to plant glycoproteins, as shown in **Figure 2**, it can be considered that these complex type FNGs must be released from matured and secreted glycoproteins fully processed in the Golgi apparatus, but not from the misfolded glycoproteins that are fated to be degraded in the cytosol. Plant specific aPNGase involved in the production of plant complex type FNGs is genetically different from cPNGase localized in the cytosol and has the optimum activity in the acidic environment. The occurrence of two subtypes of GN2-FNGs, with the truncated structure and large size structure as shown in **Figure 3**, suggests that there may be two kinds of aPNGase in plants; one works in the vacuole and the other in the extracellular space such as cell wall or the apoplast.

As described above, the structural features of FNGs found in plants indicate their origins (misfolded proteins in ER or fully processed and secreted proteins) and the enzyme, PNGase or ENGase, that is involved in the final stage of the FNGs-production. For example, structural analysis of FNGs found in cultured rice cells and culture broth provided valuable information on the deglycosylation mechanism in plant cells (Maeda et al., 2010). In the soluble fraction (a mixture of cytosolic and vacuolar fractions) of cultured cells, only high-mannose type GN1-FNGs and truncated complex type GN2-FNGs were found, suggesting that the former FNGs were produced by ENGase and the latter by aPNGase. It is believed that the plant complex type *N*-glycans linked to vacuolar-targeting glycoproteins are modified to the truncated type structure in the vacuole (Vitale and Chrispeels, 1984; Lerouge et al., 1998), and it is reasonable to assume that truncated complex type FNGs must be produced from deteriorated glycoproteins (or glycopeptides generated by endopeptidases) by vacuolar PNGase. On the other hand, high-mannose type GN2-FNGs (M_9-5_GN_2_) and two longer complex type FNGs having *N*-acetylglucosamine residue(s) or Le^a^ unit(s) at the non-reducing end (GN1 and GN2 species) have been identified from the culture broth (**Figure 3B**; Maeda et al., 2010). The occurrence of GN2-FNGs (both high-mannose type and longer complex type structures) in the culture broth suggests that the secreted-type aPNGase in either the cell wall or apoplastic area plays a critical role in the release of *N*-glycans in the extracellular space. In the structural analysis of rice FNGs, some plant complex type FNGs with unique structural feature were found in the culture broth from rice cell (Maeda et al., 2010). As shown in **Figure 3B**, the unusual FNGs carry the complex type structure at their non-reducing end and only one GlcNAc residue at their reducing end, suggesting that these FNGs were fully processed in the Golgi apparatus and the ENGase were involved in the process. As described previously, ENGases that have been purified and characterized to date cannot hydrolyze the glycosidic linkage of the *N-*acetylchitobiosyl unit of plant complex type *N-*glycans, and no other ENGase that is active against plant complex type *N-*glycans has been found. In summary, the occurrence of the plant complex type GN1-FNGs cannot be explained by the established scheme of deglycosylation and processing mechanism of *N-*glycoproteins. On the other hand, in the case of animal cells, the complex type GN1-FNGs were recently found in the stomach cancer cultured cells (Ishizuka et al., 2008), suggesting that the occurrence of complex type GN1-FNGs is not limited to plants. However, the animal complex type *N-*glycans usually carry α1-6 fucosyl (but not α1-3 fucosyl) residue linked to the innermost GlcNAc residue, and this type of animal complex type glycans can be a modest substrate for some types of ENGase. At present, therefore, the possibility of involvement of ENGase in the production of complex type GN1-FNGs in animal cells cannot be excluded.

**FIGURE 4:**
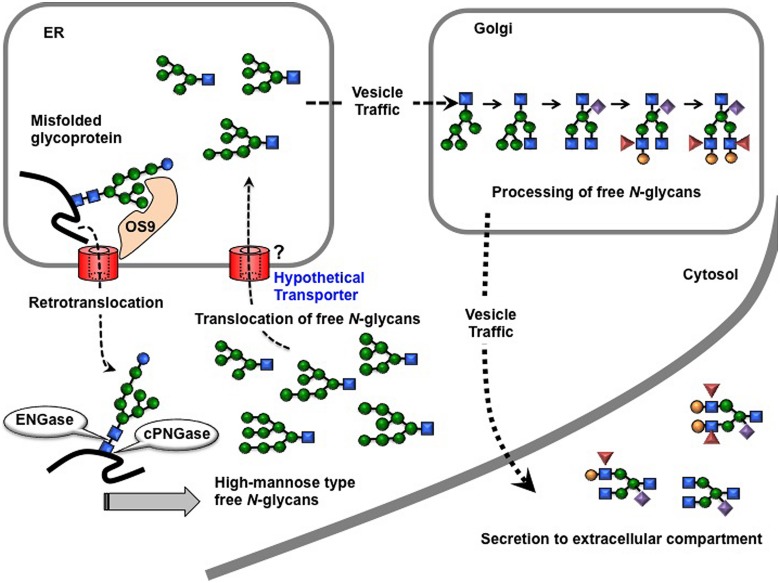
**Schematic representation of the putative processing and secretion pathway of plant complex type GN1-FNGs**. Retrotransporter for the high-mannose type GN1-FNGs produced in the cytosol has not been identified.

A putative processing scheme for the formation of the unusual FNGs, the plant complex type GN1-FNGs, has been proposed on the basis of their chemical structure (**Figure 4**), the substrate specificity of plant ENGase, and the *N-*glycan processing mechanism in the ER and Golgi apparatus (Maeda et al., 2010). Prior to discussion of the production mechanism and physiological functions of plant complex type FNGs, we would like to describe some properties of plant ENGase and PNGase, including localization, substrate specificity, and gene structure, in the following section.

## FUNCTIONAL CHARACTERIZATION OF CYTOSOLIC PNGase, ACIDIC PNGase, AND ENGase IN PLANTS

### CYTOSOLIC PNGase

Cytosolic PNGases in budding yeast (Suzuki et al., 2000), nematodes (Kato et al., 2002), and mammals (Suzuki et al., 2002) are well characterized and their gene structures identified. It is widely accepted that these cPNGase are localized in the cytosol and involved in the release of high-mannose type *N-*glycans from misfolded glycoproteins found in the ER. These cPNGase have the optimum activity in the neutral pH region and are active against denatured *N-*glycoproteins and *N-*glycopeptides bearing both high-mannose and animal complex type structures. At the same time, the glycosylated Asn residues are converted to Asp residues by the action of PNGase. It is noteworthy, however, that animal complex type *N-*glycoproteins cannot be substrates *in vivo* (or cPNGase cannot encounter these glycoproteins in cells), because cPNGase resides in the cytosol and *N-*glycoproteins carrying complex type *N-*glycans are secreted to the extracellular space through the ER and Golgi apparatus. In the case of de-*N-*glycosylation of misfolded glycoprotein in animal cytosol, the misfolded glycoproteins that retrotranslocated to the cytosol from the ER are first ubiquitinated by lectin-ubiquitin ligase Fbx2 complex, and then deglycosylated by cPNGase associated with 26S proteasome through HR23B (Rad23p in budding yeast; Suzuki et al., 2001, 2007). The resulting deglycosylated misfolded proteins are then degraded in the cytosolic proteasome complex. In contrast, the role of plant cPNGase involved in deglycosylation mechanism functioning in plant cells remains to be fully elucidated. The *Arabidopsis* cPNGase gene was identified (Diepold et al., 2007), and the cPNGase activity was recently confirmed by the transformation of the putative plant cPNGase gene into a mutant yeast strain lacking its own PNGase activity (Masahara-Negishi et al., 2012). In the transformed mutant yeast, the degradation of ERAD substrates (or misfolded glycoproteins) was facilitated in the *N-*glycan-dependent manner, indicating that the product of the putative cPNGase gene was involved in the de-*N-*glycosylation of misfolded glycoproteins. However, cPNGase activity has not been directly detected *in vitro* using misfolded glycoproteins or glycopeptides bearing high-mannose type *N-*glycans as substrates. Given that the cPNGases purified from animals or yeast can easily release the *N-*glycans from denatured *N-*glycoproteins or *N-*glycopeptides *in vitro*, the plant cPNGase may require some other specific factors to display the full amidase activity.

### PLANT SPECIFIC ACID PNGase

**FIGURE 5:**
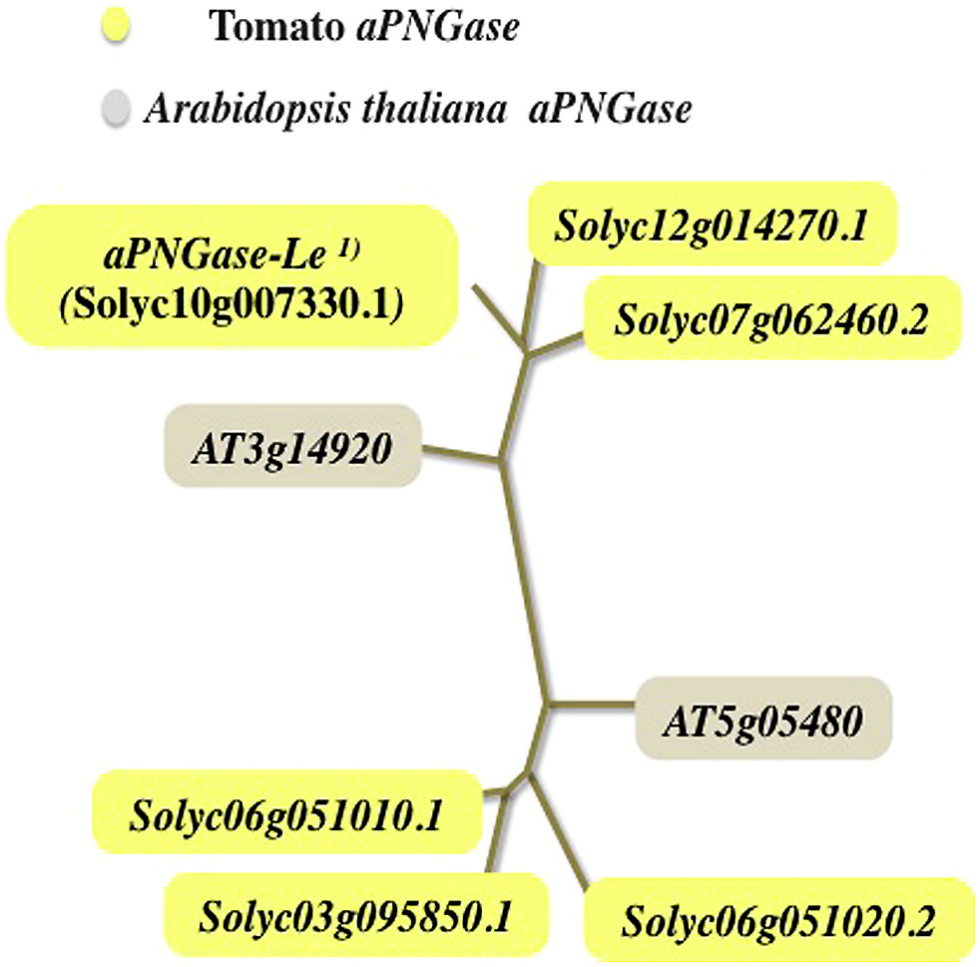
**Phylogenic tree of the acidic PNGase (tomato and *A. thaliana*): two genetic groups of plant aPNGase**. ^1)^aPNGase-Le; tomato acidic PNGase (Hossain et al., 2010b).

It is well known that another species of PNGase occurs in plants, and this kind of PNGase (aPNGase) was first purified and characterized from almond seeds (Takahashi, 1977; Takahashi and Nishibe, 1978). The plant aPNGase has the optimum activity in the acidic pH region and is active against glycopeptides carrying high-mannose type or plant complex type *N-*glycans (Takahashi, 1977; Takahashi and Nishibe, 1978; Lhernould et al., 1995; Altmann et al., 1998; Kimura and Ohno, 1998; Chang et al., 2000). However, both native *N-*glycoproteins and denatured *N-*glycoproteins are not suitable substrates for the aPNGase, suggesting that the plant aPNGase may play a role in the release of *N-*glycans from glycopeptides generated by the proteolysis of denatured glycoproteins in acidic environment such as vacuole or extracellular space. In 2010, a gene encoding tomato aPNGase was identified and the gene product when expressed in yeast showed the typical aPNGase activity (Hossain et al., 2010b). Since the orthologs of the tomato aPNGase gene were found among plants and fungi but not in animals, aPNGase must have certain specific physiological function in the development or proliferation of plants and fungi. To elucidate the plant physiological role of aPNGase, construction of transgenic plants in which the aPNGase gene is suppressed or overexpressed is necessary as the first step. Furthermore, the localization of aPNGase has not been clarified to date. Since the plant complex type FNGs (GlcNAc_2_Man_3_Xyl_1_Fuc_1_GlcNAc_2_ as an example) and the truncated type FNGs (Man_3_Xyl_1_Fuc_1_GlcNAc_2_ as an example) have been found in several plant materials, it can be assumed that there may be at least two types of aPNGase; one working in the vacuole to release the truncated type *N-*glycans and the other in extracellular space to release the longer complex type glycans. Indeed, at least two genetic groups of aPNGase have been found in the tomato genome as shown in **Figure 5**. If two types of aPNGase reside in different space (vacuole and extracellular space), it is important to reveal the molecular mechanism that directs the two types of PNGase to the adequate organelle or extracellular space in plants.

### PLANT ENGase

Endo-β-*N-*acetylglucosaminidase (ENGase) is another glycoenzyme involved in the degradation pathway of misfolded glycoproteins, and hydrolyzes the β1-4 GlcNAc linkage in high-mannose type *N-*glycans linked to glycoproteins (glycopeptides) or GN2-FNGs. Bacterial or fungal ENGases have been used as tools for structural analysis of *N-*glycans over a period of years. Especially, a fungus ENGase (Endo-M, ENGase from *Mucor hiemalis*) that has high transglycosylation activity has been applied for remodeling of biological active glycoproteins or glycopeptides (Yamamoto, 2006, 2012). On the other hand, relatively recently the animal and plant ENGases were purified and genetically characterized, although it has been reported that ENGase occurs in both animals and plants. The animal ENGase was first purified and characterized from hen oviduct (Kato et al., 1997), and then the gene structures of nematode and human ENGase were identified (Kato et al., 2002; Suzuki et al., 2002). The animal ENGases as well as the fungal ENGases (Endo-M) belong to glycoside hydrolase (GH) family 85 and these eukaryotic ENGases are genetically different from actinomycetal or bacterial ENGase such as Endo-H or Endo-F (GH Family 18).

As for plant ENGase, the functional characterization and the gene identification of the plant ENGase were reported, although the ENGase activities had been detected from several plant materials. The plant ENGase was first purified and characterized from cultured rice cells and the genes of rice, tomato, and *Arabidopsis* ENGases were successively identified (Kimura, 2007; Nakamura et al., 2009; Kimura et al., 2011). Plant ENGases have molecular weight of about 70 kDa and the optimum activity at near neutral pH (∼pH 6.5). The gene structures show that plant enzymes belong to GH family 85, suggesting that they have the transglycosylation activity like Endo-M. Plant ENGases are highly active against high-mannose type *N-*glycans bearing the following trimannosyl unit, Manα1–2Manα1–3Man1β-, such as Man_9-6_GlcANc_2_, but the activity dramatically decreases against the core structure of the high-mannose type structure (Manα1-6(Manα1-3)Manα1-6(Manα1-3)Manβ1-4GlcNAcβ1-4GlcNAc) lacking in α1-2 Man residue. The plant complex type *N-*glycans bearing β1-2Xyl and/or α1-3Fuc residues cannot be a substrate for plant ENGase, suggesting that plant ENGase is involved in the degradation mechanism of misfolded glycoproteins carrying exclusively high-mannose type *N-*glycans in the cytosol. Subcellular localization analysis using ultracentrifugation and immunofluorescence techniques have revealed that plant ENGase resides primarily in the cytosol (Kimura, 2007; Kimura et al., 2002). However, when the subcellular localization was analyzed by ultracentrifugation technique (Kimura et al., 2002), the ENGase activity was slightly detected in the ER fraction, suggesting that small amount of the ENGase may reside in the ER or ENGase in the cytosol and may interact weakly with the cytosolic side of the ER membrane. As described earlier, almost all high-mannose type FNGs in plants belong to GN1 type, indicating that the reaction of plant ENGase against high-mannose type GN2-FNGs (cPNGase products) or misfolded glycoproteins carrying high-mannose type *N-*glycans must be highly efficient.

## BIOLOGICAL FUNCTIONS OF FREE *N-*GLYCANS IN PLANTS AS SIGNALING MOLECULES

In the 1990s, auxin-like activity of plant FNGs to stimulate elongation of stems in seedlings or maturation of tomato fruit was proposed (Priem et al., 1990a; Priem and Gross, 1992; Yunovitz and Gross, 1994). As part of the study to elucidate the physiological function of FNGs involved in fruit maturation process, Nakamura et al. analyzed changes in the amount of FNGs, ENGase activities, and ENGase gene expression during tomato fruit maturation (Nakamura et al., 2008, 2009). They found that the amount of high-mannose type GN1-FNGs increased significantly with fruit maturation (mature green, breaker, pink, and mature red), but the enzyme activity (as total activity) and gene expression were nearly unchanged. These results suggested that tomato ENGase is constantly expressed during tomato fruit maturation step to produce high-mannose type GN1-FNGs. However, the amount of substrates (GN2-FNGs or misfolded glycoproteins) may increase or α-mannosidase activity (Hossain et al., 2010a; Meli et al., 2010) responsible for degradation of the GN1-FNGs may decrease. To examine the putative auxin-like activity to stimulate plant development, the transgenic *A. thaliana* plants, in which two ENGase genes were knocked out, were constructed (Kimura et al., 2011; Fischl et al., 2011). The high-mannose type GN1-FNGs found predominately in the wild plants were completely converted to GN2-FNGs in the double knockout plants and the ENGase activity was completely lost in the mutant plants, clearly indicating that plant cPNGase, as well as the animal cPNGase, is involved in the production of FNGs prior to the action of ENGase. Two single knockout (*At3g11040* or *At5g05460*) plants produced the high-mannose type GN1-FNGs, and the ENGase activities found in two single knockout plants and wild plants were comparable to each other, but the structural features of FNGs found in these single knockout mutants were slightly different between each other, suggesting that these two ENGases (At3g11040 or At5g05460) may reside in the cytosol of different tissues (roots, stems, seeds, or leaves). No apparent morphological changes were observed among the wild-type plants, two single knockout lines, and double knockout plants under the normal cultivation conditions (Fischl et al., 2011; Kimura et al., 2011). The construction of double knockout plants, in which both ENGase and cPNGase genes are completely suppressed, is the prerequisite to elucidate physiological function(s) of FNGs involved in plant differentiation, growth, and fruit maturation.

## PUTATIVE MECHANISM RESPONSIBLE FOR GENERATION OF THE PLANT COMPLEX TYPE GN1-FNGs

As described previously, the plant complex type FNGs bearing one GlcNAc residue (GN1-FNGs) at their reducing ends were first found as secreted FNGs in the rice-cell culture broth. Since these FNGs were not the truncated type and some of them carried the Le^a^ epitope, these FNGs must have been fully processed in the Golgi apparatus and secreted to extracellular space (but not sorted to the vacuole) through the trans Golgi network. If special ENGase that are active against plant complex type *N-*glycans would occur in the plant extracellular space, the occurrence of such kind of FNGs (plant complex type GN1-FNGs) could be reasonably explained. However, such special ENGase activity has not been found in plants to date. Considering the structural features of the unique GN1-FNGs, subcellular localization of ENGase and PNGase, and *N-*glycan processing pathway, a putative mechanism of the unique GN1-FNGs has been proposed (**Figure 4**; Maeda et al., 2010). In this scheme, it has been assumed that (1) the high-mannose type GN1-FNGs may be first formed by a combination of cPNGase and ENGase in the cytosol, (2) some part of the resulting GN1-FNGs may be transported from the cytosol to the ER through a putative translocator (similar to TAP, one of ABC transporters involved in the cellular immune system) and transported to the Golgi apparatus, and then (3) the high-mannose type *N-*glycans may be processed to the longer plant complex type structures and secreted to the extracellular space together with fully folded and processed proteins.

It is noteworthy to consider another interesting function of *N-*glycan involved in protein folding or reconstruction of oligomeric proteins. In a previous study (Kimura et al., 1999), it was reported that high-mannose type *N-*glycans (Man_9_GlcNAc_2_ or Glc1Man_9_GlcNAc_2_) linked to the jack bean α-mannosidase stimulated or induced the reconstruction from denatured and dissociated structure to functional oligomeric structure to recover the enzyme activity. Furthermore, Kimura et al. (1998) and Jitsuhara et al. (2002) reported that glycosylated Asn (Asn-glycan) functioned as a kind of molecular chaperon to reconstruct the functional conformation of some enzymes from denatured state, suggesting that at least the high-mannose type FNGs can play a critical role in the protein-refolding. Since it is well known that sugar molecules or polyethylene glycol stabilize protein structure and prevent protein aggregations, it is not a total surprise that the FNGs produced from glycoproteins show similar function. On the other hand, in the context of generation and degradation mechanisms of FNGs accepted to date, it is difficult to assume that unfolded or prefolded proteins in the ER encounter the high-mannose type FNGs that are formed in the cytosol. However, if there is a possibility that the high-mannose type FNGs produced in the cytosol can be transported to the ER, as postulated above, the unfolded or premature proteins, both glycoproteins and non-glycoproteins, in the ER may be able to receive the sweet benefit from the free sugar chains holding the latent chaperon-like function. To prove the hypothetical chaperon-like function of FNGs, it must be essential to confirm the occurrence of FNGs in the ER and identify the putative transporter responsible for the translocation of FNGs. Furthermore, on the platform of the biological activity of FNGs involved in the protein folding, it will be possible to develop a new glyco-technology in which FNGs are used to promote refolding of recombinant proteins *in vitro*.

## Conflict of Interest Statement

The authors declare that the research was conducted in the absence of any commercial or financial relationships that could be construed as a potential conflict of interest.
